# Hydroxychloroquine-Induced Cardiomyopathy in a Young Female With Systemic Lupus Erythematosus: A Case Report

**DOI:** 10.7759/cureus.83906

**Published:** 2025-05-11

**Authors:** Pranathi Bandarupalli, Vineeth Potluri, Shudipan Chakraborty, Harsharn Grewal, Amr Alemairy

**Affiliations:** 1 Internal Medicine, Mercy Health St. Vincent Medical Center, Toledo, USA

**Keywords:** autoimmune disorder, cardiomyopathy, drug-induced abnormalities, guideline-directed medical therapy (gdmt), hydroxychloroquine (hcq), non-ischaemic cardiomyopathy, sle (systemic lupus erythematosus)

## Abstract

Hydroxychloroquine (HCQ) is widely used in the treatment of systemic lupus erythematosus (SLE) and other autoimmune diseases due to its immunomodulatory and anti-inflammatory properties. Although generally well-tolerated, rare cases of HCQ-induced cardiomyopathy have been reported, often leading to irreversible cardiac dysfunction. We present a case of a 21-year-old female with SLE and biopsy-proven class II lupus nephritis who developed reversible cardiomyopathy associated with HCQ use. Prompt discontinuation of HCQ and initiation of guideline-directed medical therapy (GDMT) resulted in normalization of cardiac function. This case underscores the importance of early recognition and management of HCQ-induced cardiomyopathy, as well as the need for close cardiovascular monitoring in patients on long-term HCQ therapy.

## Introduction

Hydroxychloroquine (HCQ) is a cornerstone therapy for systemic lupus erythematosus (SLE) due to its ability to reduce disease activity and prevent flares [1]. Despite its favorable safety profile, prolonged use has been associated with rare but serious adverse effects, including cardiotoxicity. HCQ-induced cardiomyopathy is characterized by ventricular dysfunction, conduction abnormalities, and myocardial fibrosis, often identified by imaging or biopsy [2]. HCQ-induced cardiotoxicity is believed to result from lysosomal dysfunction and impaired autophagy in cardiomyocytes. Although rare, it has been associated with potentially irreversible cardiac injury, with reported incidence ranging from 0.6% to 1% in long-term users. This underscores the need for clinician awareness, especially when differentiating from other cardiac pathologies in SLE patients. This condition can be challenging to diagnose due to its nonspecific presentation and overlap with other causes of cardiac dysfunction in SLE patients. We report a case of HCQ-induced cardiomyopathy in a young female with SLE, highlighting the diagnostic challenges and management strategies.

## Case presentation

A 21-year-old woman with a three-year history of SLE, biopsy-proven class II lupus nephritis, essential hypertension, and anemia of chronic disease presented to the hospital with a one-week history of progressively worsening shortness of breath and bilateral lower extremity edema. She noted that her dyspnea worsened when lying flat, although she denied chest pain. Her usual medications for lupus included HCQ 300 mg daily, mycophenolate mofetil 1 g twice daily, and prednisone 20 mg daily. She had previously declined cyclophosphamide and expressed a preference for a more limited medication regimen.

On arrival, her vital signs showed a blood pressure of 140/80 mmHg, heart rate of 90 beats per minute, respiratory rate of 18 breaths per minute, and oxygen saturation of 90% on room air. She had jugular venous distension, bibasilar crackles on lung auscultation, and bilateral pitting edema in her lower extremities. Initial laboratory studies revealed a markedly elevated N-terminal pro-B-type natriuretic peptide (pro-BNP) of 952 pg/mL, suggesting volume overload or heart failure, and a high D-dimer of 845 ng/mL. Her hemoglobin was 8.2 g/dL, reflecting her baseline anemia of chronic disease. Of note, her high-sensitivity troponin levels remained negative on serial testing, indicating a low probability of myocardial infarction. Thyroid function was normal, while ferritin was elevated at 746 ng/mL. Complement levels were low (C3 of 63 mg/dL, C4 below 8 mg/dL), and anti-dsDNA was substantially elevated at 2623 IU/mL, consistent with active lupus. Urinalysis showed neither infection nor hematuria, and her urine protein-to-creatinine ratio was 0.94. Blood cultures were negative, and both serum and urine protein electrophoresis were unremarkable (Table 1). 

**Table 1:** Relevant labs

Test	Reference Range and Units	Result	Status
BNP	<100.0 pg/mL	952	High
D-dimer	<255 ng/mL DDU	845	High
Hemoglobin	11.4-15.2 g/dL	8.2	Low
C3 Complement	86-184 mg/dL	63	Low
C4 Complement	16-47 mg/dL	<8	Low
Ds DNA	<5 IU/mL	2623	High

A chest radiograph demonstrated an enlarged cardiac silhouette, pulmonary vascular congestion, and bilateral pleural effusions. An electrocardiogram showed sinus tachycardia but no ischemic changes. Given her elevated D-dimer, a CT angiogram of the chest was performed to exclude pulmonary embolism; it was negative for emboli but confirmed severe cardiomegaly, prominent pulmonary vasculature, and a small pericardial effusion. Transthoracic echocardiography revealed a left ventricular ejection fraction (LVEF) of 25-30%, mild dilation of the left ventricle with borderline increased wall thickness, and moderate mitral and tricuspid regurgitation. The estimated right ventricular systolic pressure was 52 mmHg. This was a stark contrast to an echocardiogram obtained two months earlier, when her LVEF had been normal and no valvular abnormalities were noted.

**Figure 1:**
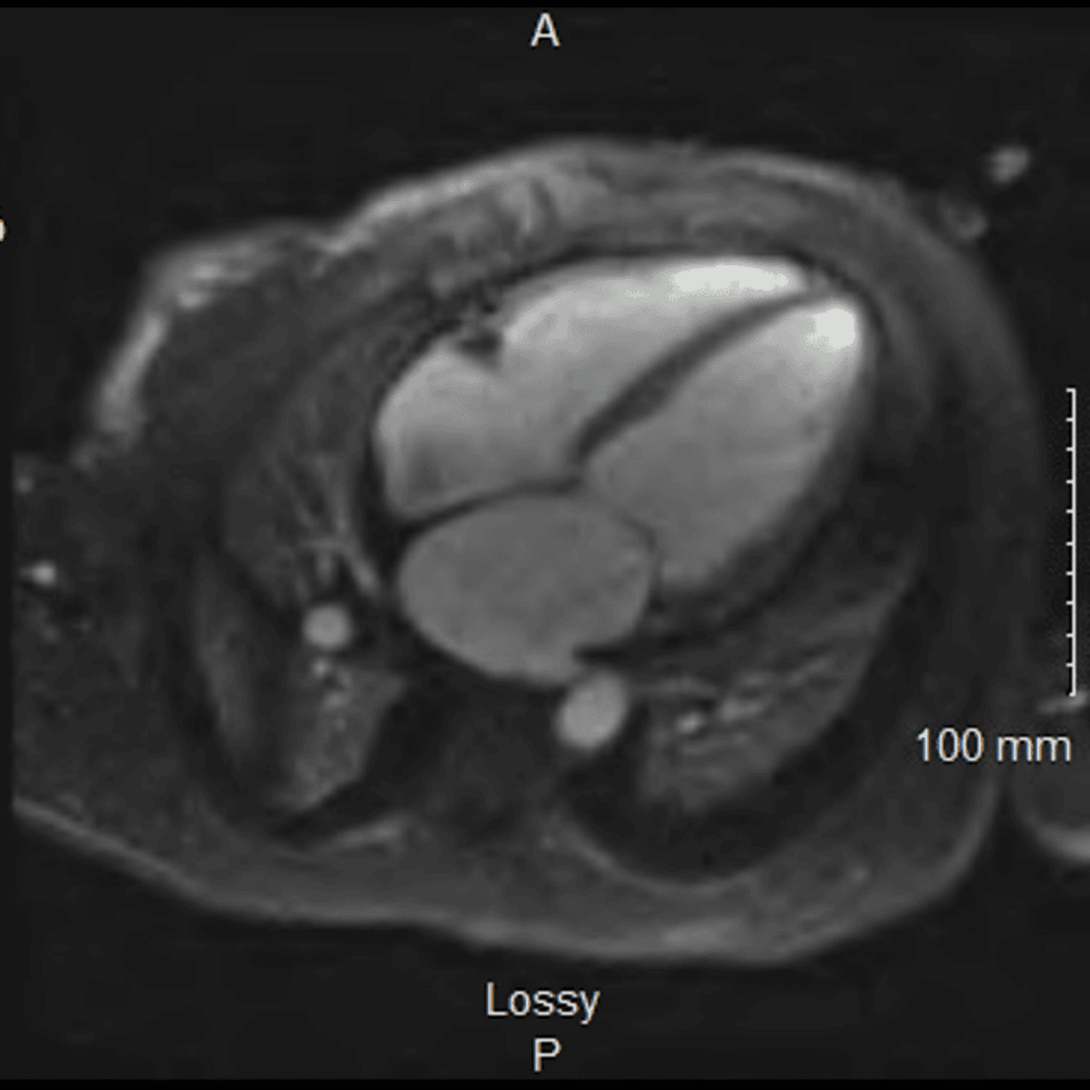
Cardiac MRI with globally diminished left ventricular systolic function, with global left ventricular hypokinesis, left ventricular dilatation, and no definitive delayed myocardial enhancement to suggest inflammatory or infectious myocarditis.

She was admitted with a working diagnosis of acute decompensated heart failure. Intravenous diuretic therapy led to a significant improvement in her volume status. Cardiac magnetic resonance imaging subsequently demonstrated an EF of 34% with subepicardial late gadolinium enhancement in the lateral wall, suggesting prior inflammatory changes but no active myocarditis or infiltrative disease (Figure 1). Although an ischemic etiology was considered, she had no chest pain and consistently negative troponins, so ischemic workup was not pursued further. There was no pericardial involvement or conduction abnormalities typically seen in lupus myocarditis. The abrupt decline in her EF, in combination with her use of HCQ, raised concern for HCQ-induced cardiomyopathy. HCQ-induced cardiomyopathy was considered a presumptive diagnosis after excluding lupus myocarditis, ischemia, and other causes. Despite elevated dsDNA and low complements, suggesting active SLE. The cardiac MRI revealed subepicardial late gadolinium enhancement in the lateral wall, without signs of active myocarditis or diffuse fibrosis. This pattern, while not pathognomonic, is more typical of resolved inflammatory processes and lacks the diffuse or patchy fibrosis seen in classic HCQ toxicity or lupus myocarditis. Troponins remained negative, and there was no pericardial involvement or conduction abnormalities typically seen in lupus myocarditis. After a multidisciplinary discussion with cardiology and rheumatology, HCQ was discontinued. She was discharged on guideline-directed medical therapy (GDMT) for heart failure, including sacubitril-valsartan, carvedilol, spironolactone, and empagliflozin, while continuing mycophenolate mofetil and prednisone for her SLE.

One month later, she returned for a follow-up. A repeat echocardiogram showed a complete recovery of her left ventricular systolic function, with an LVEF of 55-60% and only mild residual valvular regurgitation. Her shortness of breath and leg edema had resolved. This striking improvement in her cardiac function following discontinuation of HCQ strongly suggested HCQ-induced cardiomyopathy as the culprit. Her case emphasizes the importance of considering potentially reversible drug-induced causes of cardiac dysfunction in patients with autoimmune diseases and highlights the need for close collaboration among internal medicine, cardiology, and rheumatology teams.

## Discussion

HCQ remains a cornerstone in the management of SLE due to its immunomodulatory benefits and relatively safe long-term profile [1,2]. Despite these proven benefits, rare occurrences of HCQ-induced cardiotoxicity have been reported, with potential outcomes including conduction disturbances, restrictive cardiomyopathy, and dilated cardiomyopathy [3]. These presentations are often overlooked or misattributed to more common conditions affecting SLE patients, such as lupus myocarditis, ischemic heart disease, and hypertensive cardiac changes, due to overlapping clinical features.

Our case highlights this very diagnostic challenge. A young female with SLE on HCQ experienced a sudden decline in left ventricular function. Initial workup, including low troponins and the absence of chest pain or classic ECG findings, ruled out ischemic causes of cardiomyopathy. A cardiac MRI revealed no active myocarditis or infiltrative lesions, further excluding common SLE-related cardiac complications [4]. The patient’s dramatic improvement in clinical status and echocardiographic findings after HCQ discontinuation strongly implicates HCQ-induced cardiomyopathy as the cause, supported by the Naranjo algorithm for adverse drug reaction assessment [5]. These findings align with prior reports, which demonstrated that prompt withdrawal of HCQ often leads to partial or complete resolution of cardiac dysfunction [6,7]. In this case, other potential etiologies-including lupus myocarditis, ischemic cardiomyopathy, and infiltrative diseases-were ruled out through imaging, laboratory workup, and clinical correlation.

HCQ-induced cardiotoxicity is thought to arise from excessive lysosomal accumulation of the drug in cardiomyocytes, disrupting normal cellular function and autophagy [8]. Histopathological examinations have revealed vacuolar changes, myofibrillar damage, and other features akin to toxic storage cardiomyopathies [9]. While definitive diagnosis may require endomyocardial biopsy, many clinicians rely on non-invasive tools such as echocardiography, cardiac MRI, and the temporal association between drug cessation and improvement in cardiac function [8].

Distinguishing HCQ-induced cardiomyopathy from lupus myocarditis poses additional challenges, particularly in patients with active serological markers of SLE, such as elevated anti-double-stranded DNA (dsDNA) levels and low complement. However, the absence of active inflammation on cardiac MRI, combined with rapid clinical and echocardiographic improvement following HCQ discontinuation in our patient, is highly suggestive of drug-induced rather than autoimmune-mediated cardiac damage.

Based on existing literature, HCQ-induced cardiomyopathy is predominantly reported in older patients with prolonged HCQ use, often exceeding five to 10 years [10]. This case is particularly notable, as the patient was only 21 years old with approximately three years of HCQ exposure, highlighting the need for vigilance in younger populations as well. While the exact cumulative dose is unavailable, this is considered shorter than durations reported in most published cases of HCQ cardiotoxicity. Our case also suggests that susceptibility may not strictly correlate with duration. Most prior reports describe irreversible cardiac dysfunction necessitating advanced therapies such as left ventricular assist devices (LVAD). In contrast, our patient demonstrated remarkable improvement in left ventricular function with the prompt discontinuation of HCQ and initiation of GDMT, underscoring the potential for reversibility when HCQ-induced cardiomyopathy is identified early.

Certain limitations in this case warrant acknowledgment. An endomyocardial biopsy, while definitive, was not performed due to the patient’s rapid clinical improvement. Additionally, HCQ blood levels were not measured, which might have provided insight into drug accumulation and its relationship to cardiotoxicity. Although the Naranjo adverse drug reaction probability scale was applied to support HCQ as the likely etiology, we acknowledge its limitations. It is not specific to cardiotoxicity and does not account for complex autoimmune comorbidities or polypharmacy. While HCQ withdrawal is strongly temporally associated with improvement, we acknowledge that GDMT likely contributed significantly to myocardial recovery. The absence of diffuse fibrosis on imaging and rapid LVEF normalization may reflect protective factors such as early recognition, younger age, and reversible pathophysiology.

Despite these limitations, the case highlights important diagnostic and therapeutic considerations, emphasizing the need for heightened awareness among clinicians.

## Conclusions

This case underscores the importance of maintaining a high index of suspicion for HCQ-induced cardiomyopathy in SLE patients presenting with new or worsening heart failure. Vigilance is critical, regardless of age or duration of HCQ therapy, as demonstrated by this atypical presentation. Regular cardiac assessment, including periodic echocardiography, may be prudent, particularly in patients with additional risk factors such as hypertension or an atypical clinical course. Advanced imaging, particularly cardiac MRI, remains invaluable in ruling out other etiologies, such as lupus myocarditis or infiltrative cardiomyopathies, and in guiding management decisions.

Ultimately, this case demonstrates the value of a multidisciplinary approach in managing complex autoimmune conditions. Collaboration between rheumatologists, cardiologists, and other specialists fosters early recognition, precise diagnosis, and effective intervention. The patient’s recovery after HCQ cessation highlights the potential to prevent irreversible myocardial damage when drug-induced cardiotoxicity is promptly addressed. Routine cardiac screening for all HCQ users may not be justified, but targeted echocardiography - perhaps annually - could be considered in patients with pre-existing cardiovascular risk factors, high cumulative doses, or unexplained cardiopulmonary symptoms. Further research is warranted to define predictive biomarkers, validate HCQ level thresholds, and guide evidence-based screening strategies.
